# The Landscape Genetic Signature of Pollination by Trapliners: Evidence From the Tropical Herb, *Heliconia tortuosa*

**DOI:** 10.3389/fgene.2019.01206

**Published:** 2019-12-05

**Authors:** Felipe Torres-Vanegas, Adam S. Hadley, Urs G. Kormann, Frank Andrew Jones, Matthew G. Betts, Helene H. Wagner

**Affiliations:** ^1^Department of Ecology and Evolutionary Biology, University of Toronto, Mississauga, ON, Canada; ^2^Forest Biodiversity Research Network, Department of Forest Ecosystems and Society, Oregon State University, Corvallis, OR, United States; ^3^Swiss Ornithological Institute, Sempach, Switzerland; ^4^Department of Botany and Plant Pathology, Oregon State University, Corvallis, OR, United States; ^5^Smithsonian Tropical Research Institute, Panama, Panama

**Keywords:** gene flow, hummingbird, pollen pool differentiation, pollination network, pollinator recognition, TwoGener

## Abstract

Animal-mediated pollination is essential for the maintenance of plant reproduction, especially in tropical ecosystems, where pollination networks have been thought to have highly generalized structures. However, accumulating evidence suggests that not all floral visitors provide equally effective pollination services, potentially reducing the number of realized pollinators and increasing the cryptic specialization of pollination networks. Thus, there is a need to understand how different functional groups of pollinators influence pollination success. Here, we examined whether patterns of contemporary pollen-mediated gene flow in *Heliconia tortuosa* are consistent with the foraging strategy of its territorial or traplining hummingbird pollinators. Territorial hummingbirds defend clumps of flowers and are expected to transfer pollen locally. In contrast, traplining hummingbirds forage across longer distances, thereby increasing pollen flow among forest fragments, and are thought to repeatedly visit particular plants. If trapliners indeed visit the same plants repeatedly along their regular routes, this could lead to a situation where neighboring plants sample genetically distinct pollen pools. To test this hypothesis, we genotyped 720 seeds and 71 mother plants from 18 forest fragments at 11 microsatellite loci. We performed TwoGener analysis to test pollen pool differentiation within sites (among neighboring plants within the same forest fragment: *Φ* *_SC_*) and between sites (among forest fragments: *Φ* *_CT_*). We found strong, statistically significant pollen pool differentiation among neighboring mother plants (*Φ* *_SC_* = 0.0506), and weaker, statistically significant differentiation among sites (*Φ* *_CT_* = 0.0285). We interpret this pattern of hierarchical pollen pool differentiation as the landscape genetic signature of the foraging strategy of traplining hummingbirds, where repeatable, long-distance, and high-fidelity routes transfer pollen among particular plants. Although *H. tortuosa* is also visited by territorial hummingbirds, our results suggest that these pollinators do not contribute substantially to successful pollination, highlighting differences in realized pollination efficiency. This cryptic reduction in the number of realized pollinators potentially increases the vulnerability of pollination success to the decline of populations of traplining hummingbirds, which have been shown to be sensitive to forest fragmentation. We conclude that maintaining habitat connectivity to sustain the foraging routes of trapliners may be essential for the maintenance of pollen-mediated gene flow in human-modified landscapes.

## Introduction

Most species of flowering plants depend on animals for successful pollination (Ollerton et al., 2011). The resulting myriad of plant-animal interactions has given rise to a great diversity of complex pollination networks (Bascompte and Jordano, 2007), particularly in tropical ecosystems (Bawa, 1990). Due to the vast biodiversity of flowering plants and animal pollinators found in these environments, tropical pollination networks have often been thought to be highly generalized (Jordano, 1987; Olesen and Jordano, 2002); that is, a single plant species can rely on multiple functional groups of floral visitors for effective pollen transfer (Waser et al., 1996; Bascompte et al., 2006). However, this view assumes that all pollinators provide an equally effective pollination service. Accumulating evidence suggests that different functional groups of floral visitors vary widely in their effectiveness as pollinators (Waser et al., 1996; Fenster et al., 2004), leading to a reduction in the number of realized pollinators and an increase in the degree of plant-pollinator specialization (Pellmyr and Thompson, 1996; Schleuning et al., 2016). This “cryptic specialization” may increase the susceptibility of pollination to the decline of particular species of pollinators. In the face of ongoing pollination declines (Potts et al., 2010; Hadley and Betts, 2012; González-Varo et al., 2013), it is imperative to understand how different functional groups of pollinators contribute to pollination success.

Different pollinator functional traits like foraging strategy and body morphology have been shown to influence pollen flow (Ghazoul, 2005; Greenleaf et al., 2007) and pollination success (Fenster et al., 2004; Castilla et al., 2017). For example, large-bodied pollinators that forage across long distances are expected to deliver genetically diverse (“high-quality”) pollen from multiple sources and potentially increase outcrossing rates (Greenleaf et al., 2007; Ohashi and Thomson, 2009). In contrast, their small-bodied counterparts that exhibit restricted foraging patterns are thought to transfer pollen across short distances (but see Castilla et al., 2017; O’Connell et al., 2018), potentially increasing local pollination and selfing rates (Collevatti et al., 2001; Sebbenn et al., 2011). According to commonly held perspectives, large-bodied, long-distance pollinators may be more efficient at transferring high-quality pollen and contributing to successful pollination (Ohashi and Thomson, 2009) but are also highly vulnerable to habitat loss and fragmentation (Clavel et al., 2011; Aizen et al., 2012). Therefore, pollination networks with few species of realized pollinators may be more vulnerable to habitat loss and fragmentation than originally suggested by their generalized structure (Dalsgaard et al., 2008; Betts et al., 2015).

While several studies have examined the ecological consequences of increased cryptic specialization in pollination networks and their vulnerability to landscape change (Spiesman and Inouye, 2013; Hadley et al., 2014; Betts et al., 2015), there is a great need to understand how particular groups of pollinators with distinct functional traits influence pollen-mediated gene flow and pollination success (Ward et al., 2005; Castilla et al., 2017; O’Connell et al., 2018). Since pollen flow is the most prevalent avenue of gene flow in most plant species (Ennos, 1994), conserving pollen-mediated gene flow, and its resulting genetic diversity, is paramount for the long-term maintenance of pollination as an ecosystem service (Kearns et al., 1998). Moreover, maintaining pollen flow is crucial for long-term population viability (Hughes et al., 2008), as reduced pollination success can reduce seed set and potentially increase risk of population collapse (Hadley et al., 2014).

**Figure 1:**
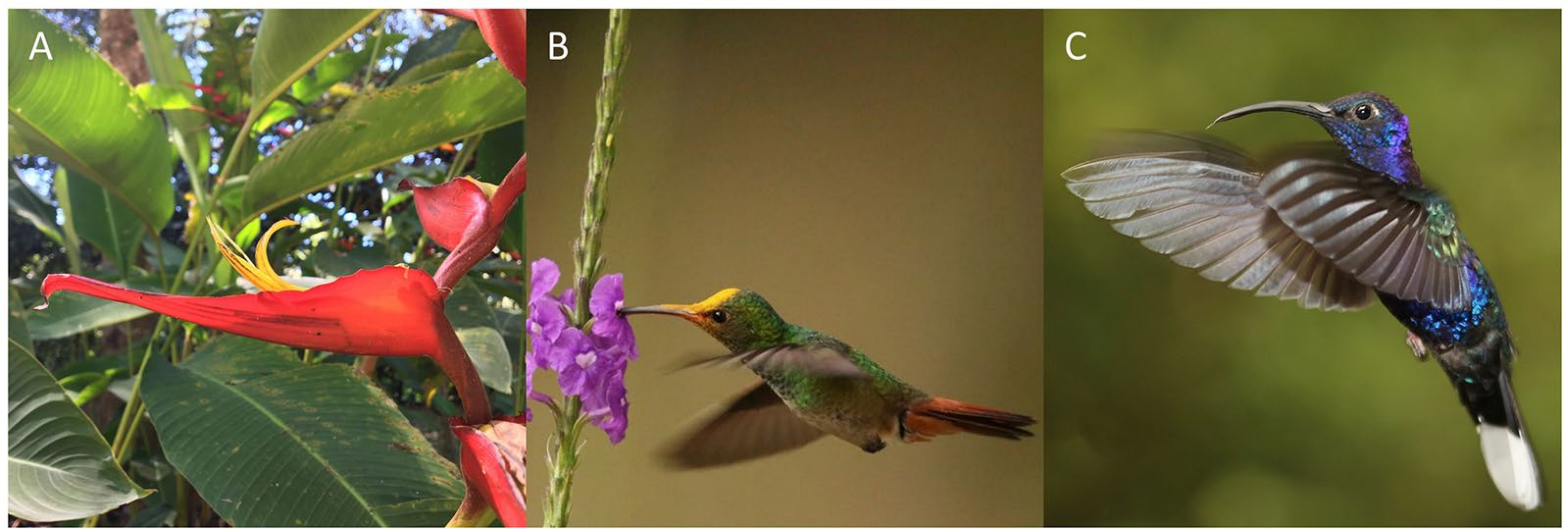
**(A)** Inflorescence of *Heliconia tortuosa*. Focus on a single red bract subtending a curved and tubular yellow flower. **(B)** A territorial rufous tailed hummingbird (*Amazilia tzacatl*). **(C)** A traplining violet sabrewing hummingbird (*Campylopterus hemileucurus*). Photo credits: Felipe Torres-Vanegas **(A)**, Matthew G. Betts **(B)**, and Urs G. Kormann **(C)**.

**Figure 2:**
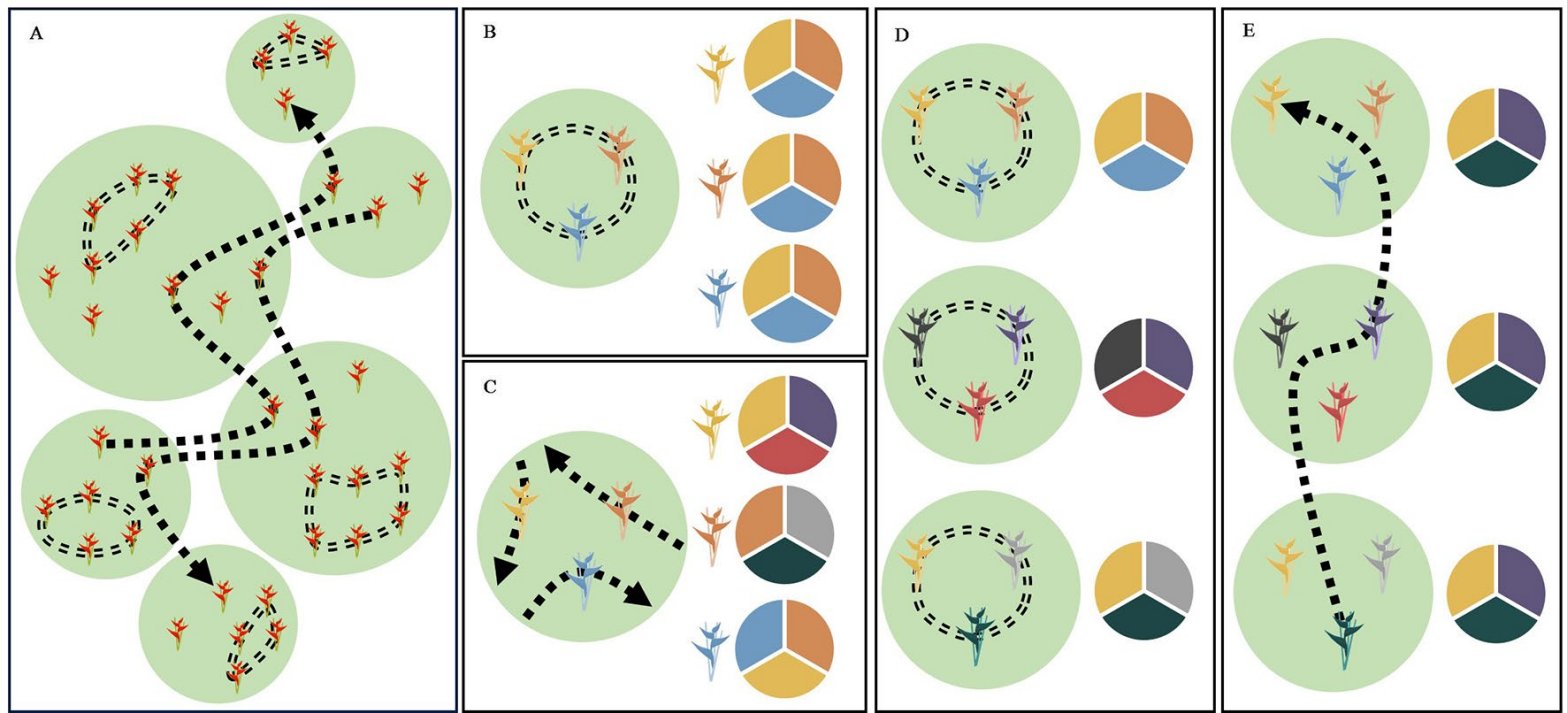
Schematic diagrams of the foraging strategies of territorial and traplining hummingbirds and their expected effect on the differentiation of pollen pools sampled among forest fragments and among neighboring plants within forest fragments. Suitable habitat for both *H. tortuosa* and its pollinators is represented in green with the matrix shown in white. **(A)** The foraging strategy of territorial hummingbirds is shown by a double-dashed line. These pollinators enhance pollen transfer among neighboring plants and reduce pollen flow among forest fragments. The foraging strategy of traplining hummingbirds is shown by a single-dashed line. These pollinators reduce pollen flow among neighboring plants and enhance pollen transfer among forest fragments. **(B)** Pollination by territorial hummingbirds leads to neighboring plants sampling the same local pollen pool, as these pollinators transfer pollen among plants within their territory and often visit multiple flowers within a clump of inflorescences. This leads to non-significant differentiation of pollen pools sampled by neighboring plants. **(C)** Pollination by traplining hummingbirds leads to neighboring plants sampling distinct pollen pools, as these pollinators will visit particular plants along different high-fidelity routes. This leads to significant differentiation of pollen pools sampled by neighboring plants. **(D)** Pollination by territorial hummingbirds reduces pollen flow among forest fragments, increasing pollen pool differentiation among sites. **(E)** Long-distance pollen transfer by traplining hummingbirds enhances pollen flow among forest fragments, reducing pollen pool differentiation among sites. Allele frequencies in pollen pools are represented by pie charts, where each color indicates a different allele.

In this study, we examined whether patterns of contemporary pollen-mediated gene flow in a tropical plant species reflect a specific pollinator foraging strategy. Our focal species, *Heliconia tortuosa* (**Figure 1A**), is an understory herb that is almost exclusively pollinated by hummingbirds that fall into two functional groups (Stiles, 1975; Betts et al., 2015). Territorial hummingbirds are small and short-billed pollinators (**Figure 1B**) that forage among nearby flowering resources. These pollinators aggressively defend small territories containing a high density of nectar resources that closely match their daily energetic requirements (Dobkin, 1984). This strategy results in restricted movement patterns (< 100 m) that constrain pollen flow (Linhart, 1987). As a result, territorial hummingbirds potentially transport pollen among neighboring or nearby individuals, reducing pollen flow among forest fragments and increasing the likelihood of inbreeding and selfing events (Hadley et al., 2017) (**Figure 2A**). Under this scenario, neighboring plants are expected to sample the same local pollen pool (**Figure 2B**), while different forest fragments are expected to sample pollen pools that are more distinct (**Figure 2D**).

In contrast, traplining hummingbirds are larger and long-billed pollinators (**Figure 1C**) that move longer distances across the landscape to acquire nectar resources and meet their daily energy expenditures (Dobkin, 1984; Gill, 1988; Ohashi and Thomson, 2009) (**Figure 2A**). These species, which can have home ranges of up to 6-ha (Volpe et al., 2014; Volpe et al., 2016), are thought to forage along established routes and repeatedly visit particular plants that offer high nectar rewards (Janzen, 1971; Tello-Ramos et al., 2015). In consequence, traplining hummingbirds potentially enhance long-distance pollen transfer and reduce pollination among neighboring or nearby plants (Taylor and White, 2007; Temeles et al., 2019), as their foraging routes result in high fidelity to their “favorite” plants (Saleh and Chittka, 2007; Klein et al., 2017). Moreover, pollen transfer among neighboring plants by trapliners may be further reduced due to the aggressive behavior of territorial hummingbirds. If traplining hummingbirds indeed visit the same individual plants along long-distance repeatable routes, then neighboring plants are expected to sample distinct pollen pools (**Figure 2C**), while different forest fragments are expected to sample pollen pools that are more similar (**Figure 2E**).

Here, we test whether patterns of contemporary pollen-mediated gene flow in *H. tortuosa* show a landscape genetic signature of pollination by traplining or by territorial hummingbirds. If successful pollination in *H. tortuosa* is dominated by traplining hummingbirds that repeatedly visit specific plants, we hypothesize that pollen pools sampled among nearby plants will be distinct and thus show significant levels of differentiation (**Figure 2C**). Moreover, long-distance foraging routes will allow traplining hummingbirds to increase pollen flow across the landscape, thereby reducing the differentiation of pollen pools sampled among forest fragments (**Figure 2E**). Alternatively, if territorial hummingbirds contribute substantially to successful pollination, we hypothesize that pollen pools sampled among nearby plants will be similar and thus not show significant levels of differentiation (**Figure 2B**). Additionally, territorial hummingbirds will most likely transfer pollen locally, thereby increasing the differentiation of pollen pools sampled among forest fragments (**Figure 2D**). This study thus adds to our understanding of how different pollinator functional traits influence patterns of contemporary pollen flow (see Krauss et al., 2017; Rhodes et al., 2017; Castilla et al., 2017; O’Connell et al., 2018; Brunet et al., 2019; Valverde et al., 2019). To our knowledge, this is the first study that assesses the landscape genetic signature of different pollinator foraging strategies by comparing the differentiation of pollen pools sampled at the local and landscape scale.

## Methods

### Study Area

The study was conducted in the human-modified landscape (∼ 31,000 ha; **Supplementary Figure 1**) surrounding the Organization for Tropical Studies Las Cruces Biological Station in southern Costa Rica (8°47N, 82°57W). Originally, the landscape was covered by Pacific pre-montane forest, but after substantial deforestation that began in the 1960s, only about 40% of the landscape remains forested (Zahawi et al., 2015). The existing forested areas span an elevation gradient from 850 to 1,500 m above sea level and are mostly surrounded by pasture and agriculture (Zahawi et al., 2015).

### Study System

We selected *H. tortuosa* and its pollinator community (**Figure 1**) as our study system because this plant is commonly visited by eight territorial and two traplining hummingbird species (Hadley et al., 2014; Betts et al., 2015) and because it is one of the most common bird-pollinated (ornithophilous) plants in the study area (Hadley et al., 2014). This species is found exclusively in the understory of premontane tropical forests, occurring individually and in small clonal clumps that may present multiple inflorescences (Stiles, 1975). *H. tortuosa* has a distinct flowering peak (February–May) during which plants produce spirally-arranged inflorescences that can hold up to 12 bracts (Stiles, 1975). In turn, each bract can subtend up to 15 flowers that open sequentially and are fertile for a single day (Stiles, 1975; Kress, 1983). Upon successful pollination, *H. tortuosa* produces fleshy fruits that contain one to three seeds. A recent study across multiple forest fragments in the study area found that the proportion of seeds produced was significantly reduced by habitat fragmentation (Hadley et al., 2014).

Ecological experiments have shown that the flowers of *H. tortuosa* can recognize visits and favor pollination by traplining hummingbirds, thus discriminating against pollen brought by territorial pollinators (Betts et al., 2015). Experimental evidence showed that pollen tube growth (a proxy for pollination success) occurs almost exclusively when flowers are fully depleted of nectar, which occurs after visits by long-billed, traplining hummingbirds (Betts et al., 2015). This mechanism, termed “pollinator recognition”, enhances the specialization between *H. tortuosa* and its traplining pollinators (Betts et al., 2015). Pollination by traplining hummingbirds produces three times more pollen tubes than pollination by territorial hummingbirds (Betts et al., 2015). However, *H. tortuosa* can also reproduce clonally by rhizomatous growth (Stiles, 1975) and it is partially self-compatible, as self-fertilization has a 25% success rate (Kress, 1983).

### Study Design

We used a stratified-random sampling design to select 20 focal forest fragments that represented a gradient of forest amount within a 1 km radius and a gradient of fragment size (for details see Hadley et al., 2014). Among the focal fragments, forest amount within a 1 km radius ranged from 9 to 66%, while fragment size ranged from 0.6 to > 1, 200 ha (**Supplementary Table 1**).

Within each focal fragment, we selected *H. tortuosa* plants at a random location (“site”) sampled within 500 m of an access point (for details see Hadley et al., 2014). Starting from this sampling location, we marked the first five plants with inflorescences (“mothers”) and sampled leaf tissue. We will refer to the set of five mother plants sampled at each site as “neighboring” plants. At the end of the flowering season, we covered the inflorescences of all selected mothers with mesh bags to avoid fruit removal by frugivores. Once fruits were mature, we randomly selected two bracts on a single inflorescence of each mother and collected all fruits. However, understory disturbance and restricted access prevented us from sampling fruits in two forest fragments. Moreover, it is common in *Heliconia* for fruits to rot before becoming fully ripe or to be aborted during development. For these reasons, we were unable to collect fruits from all sampled mothers. Therefore, the final sampled materials comprised seeds from 71 mothers sampled across 18 forest fragments.

### DNA Extraction and Microsatellite Genotyping

We extracted genomic DNA from all mothers (dried leaves) and selected embryos, which were dissected from the collected seeds. On average we genotyped 10 seeds per mother, resulting in 720 seeds from 357 fruits from 71 mothers sampled across 18 forest fragments (**Supplementary Table 1**).

All DNA extractions were completed using the QIAGEN DNeasy Plant Mini Kit following the manufacturer’s protocol (QIAGEN). Samples were amplified at 11 microsatellite loci in three multiplex reactions (Multiplex A: Hac_C7, Hb_C115, Hac_D1, Hb_B9; Multiplex B: Hac_B4, Hac_B6, Hac_C114; Multiplex C: Hac_A103, Hc_C7, Hac_A116, Hc_C126). These loci were developed for *H. acuminata*, *H. bihai*, and *H. caribaea* (Cortes et al., 2009; Gowda et al., 2012) and used for *H. tortuosa* in our study area by Jones et al. (submitted).

For each sample, a 10-µl mixture containing 0.2 µm of each primer, 4.6 µl of QIAGEN Multiplex PCR Kit Mix (QIAGEN), 1.2 µl of DNase-free water, and 5–10 ng of template DNA was used for a polymerase chain reaction (PCR) amplification. PCR was performed under the following conditions: initial denaturation at 94°C for 15 min; 36 cycles of denaturation at 94°C for 1 min, annealing at 55–65°C for 1 min, and extension at 72°C for 1.5 min; followed by a final extension at 72°C for 15 min. PCR products were loaded into an ABI 3730xl Capillary Sequencer (Applied Biosystems) using GeneScan 500 LIZ (Life Technologies) size standard for fragment length analysis at the Centre for Applied Genomics (The Hospital for Sick Children, Toronto, Canada). Genotyping was completed in Gene-Marker 2.4.0 (SoftGenetics).

Microsatellite loci were tested for departures from Hardy-Weinberg Equilibrium (HWE) using exact tests based on Monte Carlo permutation of alleles in the *R* package *pegas* (Paradis, 2010). Linkage disequilibrium was tested by estimating the index of association (*IA*) and correlation (*rBarD*) between alleles using the *R* package *Poppr* (Kamvar et al., 2014). We screened all loci for null alleles following Brookfield (1996) using the *R* package *PopGenReport* (Adamack and Gruber, 2014). All tests were carried out in R 3.4.1. (R Development Core Team, 2016). In addition, we calculated the rate of impossible genotype combinations per locus found in our mother-offspring arrays.

### Mating System

We calculated outcrossing rates for the 720 seeds sampled across 18 sites using the MLTR 3.4. software (Ritland, 2002), which implements a mixed-mating system model and a multilocus, maximum likelihood approach (Ritland and Jain, 1981). We estimated the following mating system parameters: multilocus outcrossing rate (*t* *_m_*), single locus outcrossing rate (*t* *_s_*), the multilocus correlation of paternity (*r* *_p_*), the biparental inbreeding rate (*t* *_m_* *– t* *_s_*), and the single locus inbreeding coefficient of maternal plants (*F*). We used a Newton-Raphson iteration with 1,000 bootstrap replicates to calculate standard errors (SD) of the estimated mating system parameters.

### Paternity Analysis

We conducted a paternity analysis for all sampled seeds using COLONY 2.0.6.5. (Wang, 2004; Jones and Wang, 2010). This software allows for errors in genotyping and has been shown to correctly assign a greater number of paternities in comparison to other assignment programs, particularly when potential fathers are incompletely sampled (Walling et al., 2010). Stochastic error rates per locus were assessed based on the rate of impossible genotype combinations found in the mother-offspring arrays. We used the FL-PLS analysis method (combining full likelihood methods and pairwise likelihood scores) and allowed for male and female polygamy as well as inbreeding. As recommended by the program authors (Wang, 2018), paternities were assigned with a medium-length run with high likelihood precision. We conducted a single landscape-scale paternity analysis that included all sampled mothers from all 18 sites as potential fathers.

To check whether our battery of 11 microsatellite loci had enough information to discriminate among potential fathers, we calculated exclusion probabilities using the COANCESTRY software (Wang, 2011) as the probability that a randomly chosen mother is excluded as the father of an offspring (Wang, 2007). We used CERVUS 3.0.7. (Kalinowski et al., 2007) to calculate the probability of identity of our loci, *P* *_(ID)_*, as the probability that two randomly chosen individuals share the same multilocus genotype (Waits et al., 2001). Based on the paternity assignments, we identified selfed offspring using an individual probability of assignment threshold of 0.90.

### Contemporary Pollen-Mediated Gene Flow

To determine whether successful pollination in *H. tortuosa* is dominated by traplining or by territorial hummingbirds, we quantified the genetic differentiation among pollen (paternal) genotypes across multiple levels. We thus tested for significant pollen pool differentiation among sites and among mothers nested within sites by performing a hierarchical analysis of molecular variance (AMOVA; Excoffier et al., 1992) based on pollen haplotypes (TwoGener; Smouse et al., 2001).

To obtain pollen haplotypes, we subtracted the contribution of each mother from the genotypes of each of her seeds (720 pollen haplotypes) using the *minus.mom* function in the *R* package *gstudio* (Dyer, 2014). This function resolves cases where the paternal contribution is ambiguous (mother-offspring pairs share the same heterozygous genotype) by calculating the posterior paternal-maternal gametic likelihoods for each allele, given the allele frequencies in the overall pollen pool (for details see Smouse et al., 2001). Pollen pools were constructed for each site (*n* = 18) and mother (*n* = 71) by grouping pollen haplotypes accordingly. Corresponding allele frequencies were estimated using the *allele.frequencies* function in the *R* package *gstudio* (Dyer, 2014). The multilocus pollen haplotypes were coded into a pairwise squared genetic distance matrix using the *genetic.distance* function in the *R* package *gstudio* (Dyer, 2014) following Smouse and Peakall (1999). Measures of inter-individual genetic distance were based on AMOVA distances (Excoffier et al., 1992; Smouse et al., 2001)

We fitted several hierarchical AMOVA models where we tested for significant pollen pool differentiation among sites and among mothers nested within sites (*i.e*., pairwise genetic distance ∼ site / mother). Statistics of genetic differentiation (*Φ*) were estimated for each level by partitioning the total observed genetic variation in allele frequencies into within- and among-level variance components following Smouse et al. (2001) and Dyer et al. (2004). These *Φ*-statistics are analogous to *F*-statistics and provide estimates of the genetic differentiation of pollen pools sampled among sites (*Φ* *_CT_*) and among individual mothers within sites (*Φ* *_SC_*) (Smouse et al., 2001; Dyer et al., 2004; Sork et al., 2005). These values are expected to range from zero to one, though small negative values may occur in the absence of differentiation. A value of zero indicates the absence of pollen pool differentiation (identical pollen pools; *i.e.*, same allele frequencies), while a value of one denotes complete pollen pool differentiation (non-overlapping pollen pools; *i.e.*, no shared alleles) (Smouse et al., 2001).

Although we initially genotyped 720 seeds, approximately half of them corresponded to non-independent pollination events, as seeds from the same fruit may be the product of a single floral visit and sired by the same paternal plant. Increased levels of correlated paternity among seeds from the same fruit may potentially lead to spurious pollen pool differentiation and overestimation of *Φ*-statistics. Additionally, selfing events may also inflate the degree of pollen pool differentiation, especially among neighboring plants. To control for these confounding factors, we fitted a hierarchical AMOVA model that only included outcrossed offspring and a single randomly chosen seed per fruit (independent pollination events; *n* = 343) and estimated *Φ* *_CT_* and *Φ* *_SC_* values. To assess the effect of non-independent pollination events and self-fertilization on pollen pool differentiation, we fitted two additional hierarchical AMOVA models: one that included a randomly chosen seed per fruit but did not exclude selfed seeds (*n* = 357), and one that included all seeds (*n* = 720). For all models, testing for statistical significance was conducted by permuting pollen haplotypes within the hierarchical strata (Excoffier et al., 1992). Thus, pollen pool differentiation among neighboring mothers (within site differentiation) was tested by randomly permuting seeds among mothers within the same site, whereas pollen pool differentiation among sites (among site differentiation) was tested by randomly permuting families (mothers and their corresponding seeds) among sites. All AMOVA models were fitted using the *amova* function in the *R* package *pegas* (Paradis, 2010).

The presence of spatial genetic structure (SGS) and inbreeding among sampled mothers can also influence pollen pool differentiation and lead to overestimated *Φ*-statistics (Austerlitz and Smouse, 2001; Dyer et al., 2004; Sork et al., 2005). To assess the potential for confounding of pollen flow patterns with SGS found in the adult and progeny generations, we estimated *F*-statistics separately for the 71 sampled mothers and for all 720 seeds using the *R* package *hierfstat* (Goudet, 2005). In addition, we used the FSTAT 2.9.4. software (Goudet, 1995) to calculate *R* *_ST_* (Slatkin, 1995) across all sampled mothers. Potential patterns of genetic structure in the adult generation were further examined using STRUCTURE 2.3.4. (Pritchard et al., 2000). We used an admixture model with correlated allele frequencies to estimate the most likely number of genetic clusters (*K*). We evaluated *K* values ranging from 1–18 using a 100,000 generation burn-in period, 200,000 MCMC, and 15 iterations. The most likely *K* was chosen by estimating *ΔK* following Evanno et al., (2005) and results were visualized in Structure Harvester (Earl and Von Holdt, 2012).

### Simulation of Pollen Pools

The TwoGener model assumes that mother plants are sampled at short, intermediate, and large distances (Smouse et al., 2001). Given that our sampling design includes a limited number of mothers per site (up to five) and that these were not sampled at intermediate distances within each forest fragment (neighboring mothers were sampled at a single site within each forest fragment), there is a potential for overestimating pollen pool differentiation among sites and for inflated type I error rates. In addition, the small numbers of mothers per site and seeds per mother may result in low statistical power to detect pollen pool differentiation. To evaluate these limitations, we simulated pollen pool differentiation among sites and among mothers within sites to assess the type I error rates and the statistical power of the hierarchical AMOVA models we fitted (for details see **Supplementary Methods**). Simulations were completed with different levels of genetic diversity and pollen pool differentiation. As an additional precaution, since our sampling design did not meet all the assumptions for a TwoGener analysis, we refrained from further using *Φ*-statistics to estimate population genetic parameters like the number of effective fathers (*N* *_EP_*) or average distance of pollination (*δ*).Thus, our inference was limited to testing differentiation of pollen pools sampled among sites and among neighboring mothers within sites.

## Results

### Genetic Markers

The genotyping of 71 mothers and 720 seeds across 11 microsatellite loci yielded a total of 105 alleles. Loci generally showed high levels of polymorphism, as the number of alleles per locus ranged from 4 to 17 and observed heterozygosity varied between 0.14 and 0.95. All markers except Hac_D1 showed some departures from HWE, but there were no consistent departures across sites or loci (**Supplementary Table 2**). Loci did not show any significant linkage (**Supplementary Table 3**). The rate of genotype mismatches observed between our mother-offspring arrays (**Supplementary Table 4**) suggest that the presence of null alleles across all loci was not significant (see also **Supplementary Table 7**). Thus, all markers were retained.

We found low levels of inbreeding and spatial genetic structure among the 71 sampled mothers (*F* *_ST_* = 0.024, *R* *_ST_* = 0.014, *F* *_IS_* = 0.009). Assessment of *K* values following the *ΔK* method identified *K* = 2 as the most likely number of genetic populations for sampled mothers (**Supplementary Table 5**). However, *K* = 1 had the best likelihood score (*Mean LnP(K)* = −1910.7) and the plot for *K* = 2 (**Supplementary Figure 2**) showed no discernable pattern of admixture across mothers. The 720 seeds also showed low levels of spatial genetic structure (*F* *_ST_* = 0.058), but increased inbreeding (*F* *_IS_* = 0.121). Restricting the analysis to outcrossed seeds and independent pollination events (*n* = 343) showed similar results (*F* *_ST_* = 0.045; *F* *_IS_* = 0.082).

### Mating System

Outcrossing rates (*t* ± SD) for the 720 seeds were high, (*t* *_m_* = 0.927 ± 0.015; *t* *_s_* = 0.812 ± 0.021). The multilocus correlation of paternity (*r* *_p_* ± SD) was 0.094 ± 0.014, while the biparental inbreeding rate (*t* *_m_* *– t* *_s_* ± SD) was 0.115 ± 0.015. The single locus inbreeding coefficient of maternal plants (*F* ± SD) was 0.032 ± 0.025.

### Paternity Analysis

The 11 microsatellite loci provided a multilocus exclusion probability of 0.997 and a multilocus probability of identity of < 0.001 (**Supplementary Table 4**), suggesting that the loci had enough genetic information to discriminate among potential fathers and exclude clones. Paternity analysis identified 10% of the sampled seeds as selfed (72 seeds were classified as selfed while 648 seeds were classified as outcrossed).

### Contemporary Pollen-Mediated Gene Flow

To evaluate the hypothesis that neighboring mothers sample pollen pools that are genetically distinct, as expected under pollination by traplining hummingbirds, we tested for pollen pool differentiation among sites and among mothers nested within sites. For this, we fitted three hierarchical AMOVA models that differed in whether outcrossed seeds and independent pollination events were included or not, thus accounting for potential overestimation of pollen pool differentiation among neighboring mothers due to selfing and correlated paternity. The hierarchical AMOVA that included only outcrossed seeds and independent pollination events (*n* = 343; 18 sites) showed significant pollen pool differentiation among sites (*Φ* *_CT_*) and among mothers within sites (*Φ* *_SC_*) (**Table 1A**). Specifically, we found that the degree of pollen pool differentiation among mothers within sites (*Φ* *_SC_* = 0.0506, *p* < 0.001) was almost two times greater than that among sites (*Φ* *_CT_* = 0.0285, *p* < 0.001). Note that the average pollen pool differentiation among two mothers from different sites would amount to the sum of the within- and among-site components (*Φ* *_ST_* = *Φ* *_SC_* + *Φ* *_CT_*). Thus, almost two thirds of the differentiation of pollen pools sampled among mothers from different sites (*Φ* *_SC_* */Φ* *_ST_* = 0.639) was due to pollen pool differentiation among neighboring plants, while about one third (*Φ* *_CT_* */Φ* *_ST_* = 0.360) was due to pollen pool differentiation among sites.

**Table 1:** Hierarchical AMOVA models testing pollen pool differentiation among sites (*Φ* *_CT_*) and among mothers nested within sites (*Φ* *_SC_*), with corresponding *p*-values from permutation tests.

Model	Source of genetic variation	Degrees of freedom	Sums of squares	Estimated mean squares	*Φ*-statistic	*p*-value
A	Among sites	17	505.99	29.764	*Φ* *_CT_* = 0.0285	<0.001
	Among mothers within sites	53	1060.53	20.010	*Φ* *_SC_* = 0.0506	<0.001
B	Among sites	17	515.69	30.334	*Φ* *_CT_* = 0.0273	<0.001
	Among mothers within sites	53	1096.49	20.688	*Φ* *_SC_* = 0.0635	<0.001
C	Among sites	17	891.61	52.447	*Φ* *_CT_* = 0.0332	<0.001
	Among mothers within sites	53	1537.73	29.013	*Φ* *_SC_* = 0.0862	<0.001

Model A included a single randomly chosen outcrossed seed per fruit (343 seeds from 71 mothers), thus accounting for selfing and non-independent pollination events. Model B included a single randomly chosen seed per fruit (357 seeds from 71 mothers), thus accounting for non-independent pollination events but not for selfing. Model C included all 720 seeds (from 71 mothers and 357 fruits).

A second hierarchical AMOVA model, which did not exclude selfed seeds but included a single randomly chosen seed per fruit (*n* = 357; 18 sites) (**Table 1B**), resulted in increased estimates of pollen pool differentiation among mothers within sites (*Φ* *_SC_* = 0.0635, *p* < 0.001) compared to among sites (*Φ* *_CT_* = 0.0273, *p* < 0.001). Under this model, more than two thirds of the pollen pool differentiation among mothers from different sites was due to pollen pool differentiation among neighboring plants (*Φ* *_SC_* */ Φ* *_ST_* = 0.699). Similarly, a third hierarchical AMOVA model that included all seeds (*n* = 720; 18 sites) (**Table 1C**) showed an even higher degree of pollen pool differentiation among mothers within sites (*Φ* *_SC_* = 0.0862, *p* < 0.001), which was about three times greater than that among sites (*Φ* *_CT_* = 0.0332, *p* < 0.001). Under this model, more than 70% of the differentiation of pollen pools among mothers from different sites (*Φ* *_SC_* */ Φ* *_ST_* = 0.721) was due to differentiation among neighboring plants.

### Simulation of Pollen Pools

Our simulations showed that type I error rates (0.052–0.072) for pollen pool differentiation among mothers within sites were consistent with a 95% confidence interval (0.03–0.07) around the nominal significance level of *α* = 0.05 (binomial distribution with *p* = 0.05 and *n* = 500) (**Supplementary Table 6A**). This pattern held under all combinations of genetic diversity (high and low) and pollen pool differentiation among sites (absence, low, and high). However, type I error rates for pollen pool differentiation among sites (0.066–0.170) were inflated in the presence of pollen pool differentiation among mothers within sites (**Supplementary Table 6B**).

Statistical power was high (> 0.95) both for pollen pool differentiation among sites and among mothers within sites, except for simulations that combined low levels of pollen pool differentiation among sites and among mothers within sites (0.248–0.262) (**Supplementary Table 6A**). Our empirical *Φ*-statistics were most comparable to the mean *Φ*-statistics simulated for the combination of low genetic diversity, low pollen pool differentiation among sites, and high differentiation among mothers within sites (empirical *Φ* *_SC_* = 0.0506 and *Φ* *_CT_* = 0.0285; simulated *Φ* *_SC_* = 0.083 and *Φ* *_CT_* = 0.036) (**Supplementary Table 6C**).

## Discussion

### Landscape Genetic Signature of Pollination by Traplining Hummingbirds

Strikingly, we found that neighboring mothers sampled genetically differentiated pollen pools and that these were, on average, more differentiated than the local pollen pools available at sites from different forest fragments. Estimates of pollen pool differentiation among mothers within sites were about two times higher than estimates of pollen pool differentiation among sites, even after accounting for potential spurious pollen pool differentiation due to a combination of selfing (**Table 1A**) and non-independent pollination events (correlated paternity) (**Table 1B**). This pattern of hierarchical pollen pool differentiation is consistent with the foraging strategy of traplining hummingbirds (**Figures 2C, E**), but not with the movement patterns of territorial hummingbirds (**Figures 2B, D**). Specifically, we argue that pollen pool differentiation among neighboring mothers is a landscape genetic signature of the traplining foraging strategy, as it cannot be explained by the spatial scale of pollen flow alone but requires neighboring mothers to consistently receive pollen from a distinct set of fathers, such as the repeated sequence of plants or inflorescences a traplining hummingbird is expected to visit along its established foraging route (**Figure 2A**).

The foraging strategy of traplining hummingbirds has important consequences for the spatial patterns of pollen flow. Long-distance foraging patterns allow trapliners to visit multiple forest fragments during a single bout (Taylor and White, 2007; Volpe et al., 2014), likely increasing pollen flow between them and thus reducing pollen pool differentiation among sites (**Figure 2E**). Also, it is difficult for trapliners to visit multiple neighboring plants due to the aggressive behavior of territorial hummingbirds (personal field observation). Therefore, traplining hummingbirds are unlikely to contribute substantially to local pollination. Finally, the fidelity of traplining hummingbirds to specific plants or inflorescences reduces the chances that neighboring plants receive pollen from the same father, thus increasing pollen pool differentiation among mothers within sites (**Figure 2C**).

In our system, the landscape genetic signature of pollination by traplining hummingbirds may be promoted by two factors: (1) the flowering phenology of *H. tortuosa* in which a limited number of flowers per inflorescence offer fresh nectar rewards every day (Stiles, 1975; Dobkin, 1984); and (2) the presence of a pollinator recognition mechanism (Betts et al., 2015). Field experiments showed that pollen tube growth in *H. tortuosa* occurred when flowers were visited by traplining hummingbirds but was reduced significantly when visits were made by territorial hummingbirds (Betts et al., 2015). Based on these ecological experiments, we expected that traplining hummingbirds, and not territorial pollinators, would be largely responsible for successful pollination (Betts et al., 2015). We found that our estimates of pollen pool differentiation are consistent with this expectation, as the landscape genetic signature of pollination by traplining hummingbirds shown here confirms that effective pollen transfer is largely due to these species.

Although previous work in this system showed that territorial hummingbirds can contribute to pollen tube growth (Betts et al., 2015), our results suggest that these species contribute little to realized seed production. Based on their foraging strategy, territorial hummingbirds are expected to spatially restrict pollen flow (< 100 m), leading to increased pollen transfer among neighboring plants and reduced pollen flow between fragments (Hadley et al., 2017). This scenario should not increase the differentiation of pollen pools sampled by neighboring mothers (**Figure 2B**) but result in increased pollen pool differentiation among sites (**Figure 2D**). However, our results suggest the opposite, as we found high levels of pollen pool differentiation among mothers within sites and lower levels of pollen pool differentiation among sites (**Tables 1A–C**). Thus, the absence of a landscape genetic signature of pollination by territorial hummingbirds suggests that these pollinators do not contribute significantly to successful pollination in *H. tortuosa*.

Alternatively, increased pollen pool differentiation among mothers within sites could potentially arise when analyzing selfed seeds or non-independent pollination events that may increase the degree of correlated paternity between seeds from the same fruit, leading to spurious pollen pool differentiation. However, our hierarchical AMOVA model that included a single randomly chosen outcrossed seed per fruit (**Table 1A**) confirmed that even after accounting for non-independent pollination events and selfing, neighboring mothers sample pollen pools that are genetically distinct, more so than the local pollen pools sampled at different sites. This further supports the hypothesis that hierarchical patterns of pollen pool differentiation in *H. tortuosa* bear the landscape genetic signature of pollination by traplining hummingbirds. Additionally, the estimated rates of outcrossing (*t* *_m_* *, t* *_s_*), correlated paternity (*r* *_p_*), and selfing (10%) confirmed that the proportions of selfed seeds and full sibs are low, as expected under pollination by trapliners. Finally, we found no evidence of strong spatial genetic structure or inbreeding in the mother generation. Thus, we deem the potential to overestimate *Φ* statistics in our analysis to be low.

Our simulation results suggest that type I error rates for testing pollen pool differentiation among mothers nested within sites (*Φ* *_SC_*) are within the expected range. Thus, our AMOVA models that test pollen pool differentiation at this hierarchical level can be considered reliable. However, in the presence of pollen pool differentiation among mothers within sites, our simulations showed an increased type I error rate when testing pollen pool differentiation among sites (*Φ* *_CT_*). Hence, our results showing statistically significant pollen pool differentiation among sites should be interpreted with caution. Further, if pollen pools sampled by neighboring mothers show a high degree of overlap (no differentiation), the lack of mothers sampled at intermediate distances could potentially lead to overestimation of pollen pool differentiation among sites. However, since our results show that neighboring mothers sample significantly differentiated pollen pools, undersampling of pollen pools is less likely. Based on the limitation of our sampling design, future work testing pollen pool differentiation among plants pollinated by trapliners should consider a multi-stage sampling design that samples mother plants at multiple sites per forest fragment, therefore allowing further population genetic inference.

### Genetic Consequences of Pollination by Traplining Hummingbirds

Effective pollen transfer by traplining hummingbirds may have several important consequences for the maintenance of successful pollination in human-modified landscapes. First, long-distance foraging by traplining hummingbirds may facilitate the transfer of high-quality pollen from multiple sources (Ohashi and Thomson, 2009; Betts et al., 2015; Hadley et al., 2017), potentially increasing the genetic diversity of the sampled pollen pools. Thus, accepting pollen delivered by traplining hummingbirds can potentially facilitate mate selection and increase plant fitness (Hadley et al., 2014; Betts et al., 2015). We hypothesize that because of their potential to deliver high-quality pollen, traplining hummingbirds are a key determinant of pollen pool genetic diversity. Future work should examine how the genetic diversity of pollen pools may be impacted by reduced availability of traplining hummingbirds.

Second, the unequal contribution to pollination success between territorial and traplining hummingbirds substantially reduces the number of realized pollinators. Thus, successful pollination in *H. tortuosa* could be more vulnerable to landscape alteration than originally suggested by the generalized structure of the pollination network. Indeed, previous ecological research in this system showed that the availability and species richness of traplining hummingbirds decline significantly with habitat loss and fragmentation (Kormann et al., 2016; Hadley et al., 2017). Therefore, continued deforestation has the potential to reduce long-distance pollen transfer and increase the rate of local pollination events, likely increasing inbreeding and selfing (Collevatti et al., 2001; Sebbenn et al., 2011; Jones et al. submitted). Identifying and conserving large and connected forest fragments that harbor the foraging routes of traplining hummingbirds, as well as increasing the structural connectivity among isolated fragments, are two essential strategies for the maintenance of pollen-mediated gene flow in human-modified landscapes. Future work should also examine whether the contribution of territorial hummingbirds to successful pollination increases under declining availability of traplining hummingbirds.

Although our study isolated the patterns of contemporary pollen-mediated gene flow in *H. tortuosa*, the patterns of spatial genetic structure of this plant population will also be influenced by seed-mediated gene flow. To generate a complete assessment of gene flow and patterns of spatial genetic structure in *H. tortuosa*, it is important to consider this additional plant-animal interaction. This could be assessed by comparing patterns of genetic differentiation between pre-dispersal seeds and post-dispersal plant juveniles across multiple hierarchical levels (within and among forest fragments) and by evaluating how these are influenced by habitat loss and fragmentation, including altered pollinator and seed disperser communities.

### Pollinator Functional Traits and Pollination Effectiveness

Our results are consistent with other studies that examined how pollinator functional traits (body morphology, body size, foraging strategy) influence pollination success. In other systems, high morphological affinity between floral structures and pollinator characteristics have been reported to facilitate flower handling (Maglianesi et al., 2014), increase pollen removal (Watts et al., 2012; Rhodes et al., 2017; Temeles et al., 2019), and enhance the genetic diversity of sired seeds (Valverde et al., 2019). In *H. tortuosa*, flower morphology is closely matched by the long and curved bills of traplining hummingbirds. This allows trapliners to extract high amounts of nectar, thus activating the pollinator recognition mechanism and inducing pollen tube growth (Betts et al., 2015). In contrast, the short bills of territorial hummingbirds are not long enough to deplete nectar resources, reducing their importance as effective pollinators (Betts et al., 2015). Thus, we hypothesize that the landscape genetic signature of pollination by traplining hummingbirds reported here is facilitated by the close morphological fit between the bills of these pollinators and the flowers of *H. tortuosa*.

Additionally, the body size of pollinators has been shown to be positively correlated with foraging and pollen transfer distance (Greenleaf et al., 2007; Kapustjanskij et al., 2007), two traits that are thought to enhance pollination success (Ohashi and Thomson, 2009). These observations are consistent with our results, as genetic evidence presented here suggests that successful pollination in *H. tortuosa* occurs largely when flowers are visited by large-bodied pollinators (traplining hummingbirds). Although our results align with the classical assumption that large-bodied pollinators enhance pollination success, the relationship between pollinator body size, pollen transfer distance, and pollination effectiveness is difficult to generalize and has proven to be context-dependent. For example, other studies have found that small-bodied pollinators can transfer pollen just as a far as large-bodied floral visitors (Klein et al., 2017; O’Connell et al., 2018), potentially enhancing long-distance pollen-mediated gene flow and genetic diversity (Castilla et al., 2017). Thus, pollinator body size alone is an inadequate predictor of pollen transfer distance and pollination success.

Few studies have examined the genetic consequences of different pollinator foraging strategies on contemporary pollen flow. Recent efforts based on distinct pollinator taxa have shown that traplining is a learned behavior that allows pollinators to visit their “favorite” flowers in a repeatable sequence (Saleh and Chittka, 2007; Ohashi and Thomson, 2009), resulting in high fidelity to specific plants (Ohashi et al., 2008; Klein et al,. 2017) and a decrease in pollen flow among individuals that are not included in such traplines (Temeles et al., 2019). Thus, predictive models for pollen movement suggest that plants that are regularly visited by traplining hummingbirds are expected to receive pollen from multiple sources, increasing mate diversity, outcrossing rates, and pollination success (Ohashi and Thomson, 2009; Krauss et al., 2017). Further research is needed to assess whether traplining results in pollen pool differentiation among nearby plants in other systems; or whether the strong genetic signal of traplining found here is related to the presence of a pollinator recognition system, which may amplify the genetic consequences of the traplining foraging strategy.

Our results suggest that biotic interactions such as pollination, in addition to the potential effects of geographical isolation, can drive patterns of genetic differentiation among plants. Other studies of hummingbird-pollinated plants have found similar results, where pollinator habitat preferences restrict gene flow and increase genetic differentiation between plant populations (Wanderley et al., 2018). Therefore, the landscape genetic signature of pollination by trapliners highlights the importance of pollinator foraging behavior as a determinant of genetic differentiation in hummingbird-pollinated plants.

Although *H. tortuosa* is visited by multiple species of hummingbirds, our study suggests that this pollination network has high cryptic specialization (Fenster et al., 2004), where successful pollination largely comes from the few traplining hummingbird species. A reduction in the number of realized pollinators has been reported in other species of plants that are visited by multiple functional groups of pollinators (Watts et al., 2012; Rhodes et al., 2017; Valverde et al., 2019; Temeles et al., 2019). Therefore, our results support a growing body of work showing that the total number of floral visitors is neither a good predictor nor any guarantee of effective pollen transfer, especially in the Anthropocene where habitat loss and fragmentation are expected to continue (Defries et al., 2010; Hansen et al., 2013).

## Data Availability Statement

The datasets generated for this study can be found in Figshare (10.6084/m9.figshare.10108706) and Dryad (https://datadryad.org/stash/dataset/doi:10.5061/dryad.tmpg4f4tw). 

## Author Contributions

This study contributes to a larger research project initiated by AH, MB, and AJ, who collectively planned and oversaw the collection of materials by field technicians. HW and AH conceptualized the present landscape genetic study, and HW designed the simulation experiment. FT-V conducted the laboratory work, analyzed the data, and drafted the manuscript. All authors contributed substantially to the revision and editing of this manuscript.

## Funding

This research was supported through scholarships to FT-V by Colfuturo and Colciencias. Additional funding came from the Natural Sciences and Engineering Council of Canada (NSERC) through a Discovery Grant to HW, the CREATE program “ADVENT/ENVIRO” (Murray et al.), and an NSERC Postdoctoral Fellowship to AH. Field sampling was funded by two National Science Foundation (NSF) grants (NSF-DEB-1050594) to MB and (NSF-DEB-1457837) to MB, AH, and AJ.

## Conflict of Interest

The authors declare that the research was conducted in the absence of any commercial or financial relationships that could be construed as a potential conflict of interest.
