# Supplementary material for: The Landscape Genetic Signature of Pollination by Trapliners: Evidence From the Tropical Herb, Heliconia tortuosa
Source: Front Genet. 2019 Dec 5;10:1206. doi: 10.3389/fgene.2019.01206 (PMC6906188; doi:10.3389/fgene.2019.01206)
Supplement: Supplementary file 1 [file DataSheet_1.pdf]

Supplementary Material

1 Supplementary Figures

1.1 Supplementary Figure 1

Map of the study region in southern Costa Rica. Sampling locations within focal fragments (‘sites’) are represented by filled circles (n = 18). Numbers represent the ID of each focal fragment. Only focal forest fragments included in the genetic analysis are shown.

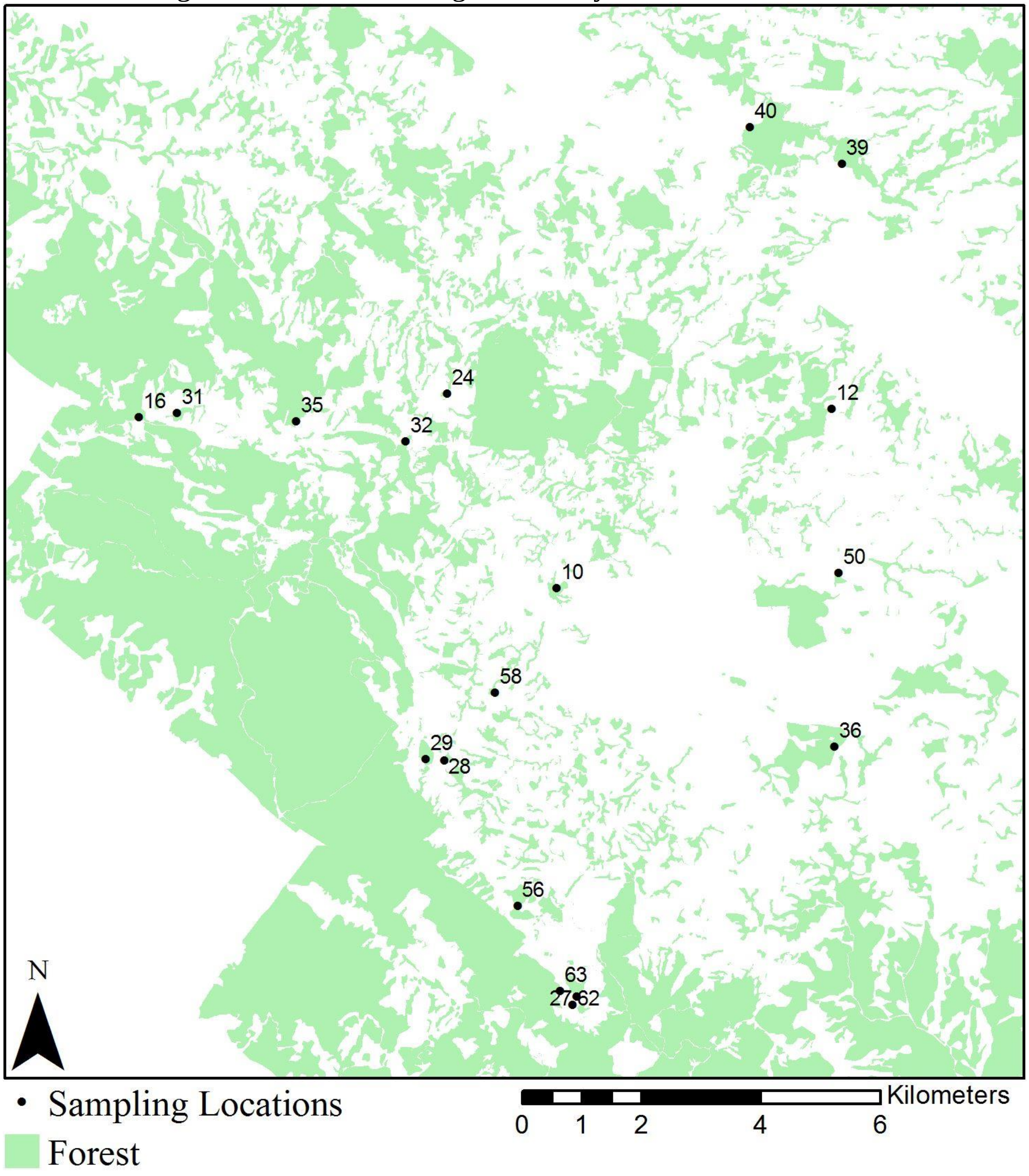

1.2 Supplementary Figure 2

Structure bar plot showing assignment probabilities ($K = 2$) for all mother plants ($n = 71$) sampled across 18 sites. The y-axis corresponds to individual probabilities of assignment. The x-axis indicates the ID of each focal forest fragment.

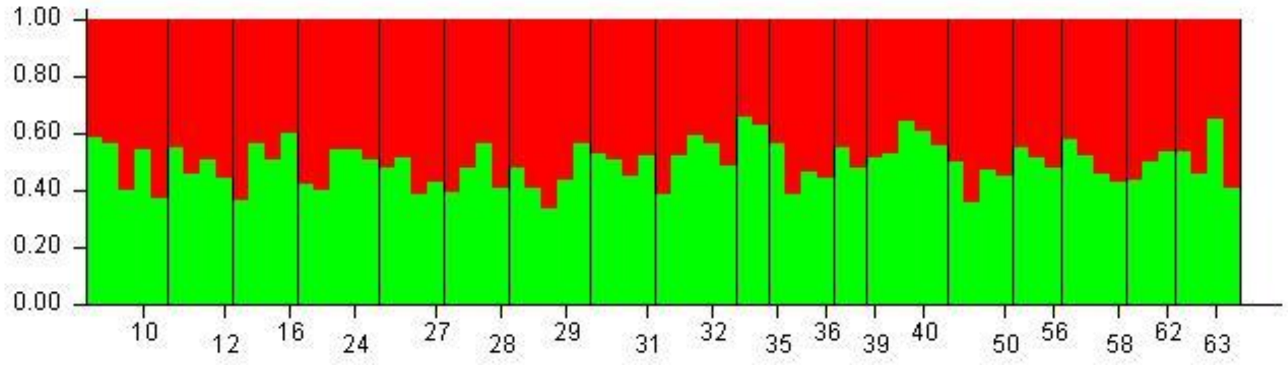

2 Supplementary Tables

2.1 Supplementary Table 1

Summary data for the focal forest fragments in our study region. Only focal forest fragments included in the genetic analysis are shown.

Focal Forest Fragment ID	Genotyped Focal Plants (Mothers)	Sampled Fruits	Genotyped Seeds	Focal Forest Size (ha)	Percent of Forest Cover Surrounding Focal Fragment (1-km)
10	5	32	54	8.93	9.83
12	4	22	42	53.91	30.71
16	4	21	47	1291.58	65.26
24	5	22	49	1.75	2.29
27	4	20	40	6.80	5.83
28	4	14	34	0.58	3101
29	5	20	47	7.19	41.50
31	4	18	40	5.62	51.48
32	5	31	51	53.90	41.96
35	2	15	29	266.82	42.40
36	4	19	38	67.88	26.89
39	2	11	22	59.86	23.70
40	5	25	48	85.85	37.00
50	4	18	37	2.97	17.23
56	3	18	32	15.68	43.92
58	4	19	41	11.98	19.65
62	3	15	32	0.68	58.90
63	4	17	37	957.26	51.11

2.2 Supplementary Table 2

Probability values testing for departure from Hardy-Weinberg Equilibrium for each combination of focal forest fragment and microsatellite marker. The *p*-values were calculated using exact tests based on Monte Carlo permutation of alleles and corrected for false discovery rates.

Focal Forest Fragment ID	Hac_C7	Hac_D1	Hb_B9	Hb_C115	Hac_C114	Hac_B6	Hac_B4	Hac_A103	Hac_A116	Hc_C7	Hc_C126
10	1.00	0.41	0.77	0.18	1.00	0.00	0.01	0.42	1.00	0.48	1.00
12	0.44	0.33	0.63	0.26	1.00	0.00	0.00	0.00	0.16	0.66	1.00
16	0.26	0.26	0.96	0.33	1.00	0.05	0.00	0.26	1.00	0.50	0.20
24	1.00	0.59	0.93	1.00	1.00	0.97	0.06	0.45	0.47	0.81	0.50
27	0.50	0.78	0.44	0.18	0.50	0.11	0.26	0.21	1.00	1.00	0.59
28	0.41	1.00	0.50	0.29	1.00	0.00	0.14	0.00	1.00	1.00	1.00
29	0.96	1.00	0.29	1.00	1.00	0.00	0.00	0.53	1.00	0.41	1.00
31	0.77	0.41	0.29	0.35	0.50	0.00	0.33	0.61	0.50	0.17	1.00
32	0.41	0.59	0.18	0.12	1.00	0.15	0.47	0.10	0.75	0.56	1.00
35	0.66	0.50	0.26	0.23	0.47	0.44	1.00	0.77	0.96	0.73	0.42
36	0.41	0.18	0.47	0.33	1.00	0.02	0.50	0.45	0.06	0.00	0.17
39	1.00	1.00	0.00	0.19	1.00	0.07	0.43	0.01	1.00	0.97	1.00
40	0.07	0.93	0.05	0.02	0.32	0.00	0.06	0.29	0.45	0.13	1.00
50	0.69	0.78	0.03	0.74	1.00	0.00	0.23	1.00	1.00	0.77	1.00
56	0.30	0.50	0.01	0.04	1.00	0.01	0.66	0.00	0.26	0.97	0.41
58	1.00	0.97	0.02	0.06	0.96	0.05	0.06	0.60	0.46	0.02	1.00
62	0.49	1.00	0.60	0.06	1.00	0.06	0.35	0.00	1.00	0.32	1.00
63	1.00	0.59	0.00	0.08	0.50	0.00	0.03	0.03	0.41	0.38	0.60

2.3 Supplementary Table 3

Overall test for linkage disequilibrium. The index of association (*IA*) and correlation (*rBarD*) between all locus pairs are shown.

Linkage Pair	*IA*	*rBarD*
Hac_C7:Hac_D1	0.01	0.01
Hac_C7:Hb_B9	0.03	0.03
Hac_C7:Hb_C115	0.04	0.04
Hac_C7:Hac_C114	0.05	0.05
Hac_C7:Hac_B6	0.01	0.01
Hac_C7:Hac_B4	0.03	0.03
Hac_C7:Hac_A103	0.00	0.00
Hac_C7:Hac_A116	0.04	0.04
Hac_C7:Hc_C7	0.04	0.04
Hac_C7:Hc_C126	-0.03	-0.03
Hac_D1:Hb_B9	0.03	0.03
Hac_D1:Hb_C115	0.01	0.01
Hac_D1:Hac_C114	0.01	0.02
Hac_D1:Hac_B6	0.00	0.00
Hac_D1:Hac_B4	0.02	0.02
Hac_D1:Hac_A103	-0.01	-0.01
Hac_D1:Hac_A116	0.01	0.01
Hac_D1:Hc_C7	-0.01	-0.01
Hac_D1:Hc_C126	0.01	0.01
Hb_B9:Hb_C115	0.10	0.10
Hb_B9:Hac_C114	0.02	0.02
Hb_B9:Hac_B6	0.01	0.01
Hb_B9:Hac_B4	0.06	0.06
Hb_B9:Hac_A103	0.03	0.03
Hb_B9:Hac_A116	-0.01	-0.02
Hb_B9:Hc_C7	0.05	0.05

Hb_B9:Hc_C126	0.02	0.02
Hb_C115:Hac_C114	0.04	0.05
Hb_C115:Hac_B6	0.02	0.02
Hb_C115:Hac_B4	0.07	0.07
Hb_C115:Hac_A103	0.09	0.09
Hb_C115:Hac_A116	0.05	0.05
Hb_C115:Hc_C7	0.08	0.08
Hb_C115:Hc_C126	-0.01	-0.01
Hac_C114:Hac_B6	0.00	0.00
Hac_C114:Hac_B4	0.04	0.04
Hac_C114:Hac_A103	0.02	0.02
Hac_C114:Hac_A116	0.03	0.03
Hac_C114:Hc_C7	0.04	0.04
Hac_C114:Hc_C126	0.00	0.00
Hac_B6:Hac_B4	0.04	0.04
Hac_B6:Hac_A103	0.04	0.04
Hac_B6:Hac_A116	0.03	0.03
Hac_B6:Hc_C7	0.02	0.02
Hac_B6:Hc_C126	0.00	-0.01
Hac_B4:Hac_A103	0.08	0.08
Hac_B4:Hac_A116	0.03	0.03
Hac_B4:Hc_C7	0.12	0.12
Hac_B4:Hc_C126	0.06	0.06
Hac_A103:Hac_A116	0.01	0.01
Hac_A103:Hc_C7	0.10	0.10
Hac_A103:Hc_C126	0.02	0.03
Hac_A116:Hc_C7	0.04	0.04
Hac_A116:Hc_C126	-0.02	-0.02
Hc_C7:Hc_C126	0.04	0.04

2.4 Supplementary Table 4

Locus information content. The observed number of alleles per locus, observed heterozygosity, expected heterozygosity, genotyping error rates, exclusion probability, and probability of identity (P_{ID}) are shown. The information content corresponds to 71 mothers and 720 seeds combined.

Locus	Number of Alleles	Observed Heterozygosity	Expected Heterozygosity	Genotyping Error Rate	Exclusion Probability	Probability of Identity
Hac_C7	8	0.42	0.49	0.01	0.262	0.313
Hac_D1	5	0.48	0.50	0.01	0.219	0.345
Hb_B9	10	0.53	0.69	0.03	0.473	0.132
Hb_B115	13	0.62	0.77	0.05	0.561	0.087
Hac_C114	4	0.14	0.15	0.00	0.074	0.724
Hac_B6	6	0.34	0.63	0.08	0.346	0.213
Hac_B4	13	0.95	0.83	0.06	0.675	0.047
Hac_A103	14	0.59	0.74	0.03	0.562	0.087
Hac_A116	9	0.24	0.27	0.00	0.155	0.537
Hc_C7	17	0.66	0.78	0.04	0.595	0.075
Hc_C126	6	0.19	0.22	0.00	0.114	0.623
All Loci	105	0.46	0.55	0.02	0.997	< 0.001

2.5 Supplementary Table 5

Evanno et al. (2005) method results for evaluation of spatial genetic structure among sampled mothers with STRUCURE. The total number of groups (*K*), iterations, mean likelihood (*Mean LnP(K)*), variance per *K* (*Stdev LnP(K)*), first order rate of change per *K* (*Ln'(K)*), second order rate of change per *K* (|*Ln''(K)*|), and delta *K* (*ΔK*) values are shown. The model with a single genetic population, *K* = 1, had the highest mean likelihood value (note that the Evanno method does not allow for testing *K* = 1).

K	Iterations	Mean LnP(K)	Stdev LnP(K)	Ln'(K)	|Ln''(K)|	Delta K
1	15	-1910.72	0.35	NA	NA	NA
2	15	-1911.26	1.48	-0.54	11.33	7.64
3	15	-1923.14	6.62	-11.87	4.54	0.68
4	15	-1939.56	10.79	-16.42	10.32	0.95
5	15	-1966.30	14.35	-26.74	2.24	0.15
6	15	-1990.80	24.64	-24.50	20.54	0.83
7	15	-1994.76	38.78	-3.96	13.49	0.34
8	15	-1985.22	39.81	9.53	2.62	0.06
9	15	-1973.06	54.91	12.16	13.06	0.23
10	15	-1973.97	44.83	-0.90	8.87	0.19
11	15	-1983.75	44.37	-9.78	31.56	0.71
12	15	-1961.96	58.16	21.78	8.29	0.14
13	15	-1948.47	39.85	13.49	16.84	0.42
14	15	-1951.82	39.51	-3.34	17.38	0.43
15	15	-1937.78	44.23	14.03	5.21	0.11
16	15	-1928.96	27.03	8.82	7.29	0.26
17	15	-1927.44	21.98	1.52	6.66	0.30
18	15	-1932.57	27.99	-5.13	NA	NA

2.6 Supplementary Table 6

(A) Approximate type I error rates (based on 500 replicate simulations) and statistical power based on simulations of differentiation of pollen pools sampled among mothers within sites. Numbers in bold denote scenarios with low statistical power.

**Test of Pollen Pool Differentiation Among Mothers Within Sites**	**Genetic Diversity**	**Pollen Pool Differentiation Among Sites**		
		Absence	Low	High
Type I error rate	High	0.058	0.072	0.054
	Low	0.054	0.052	0.058
Power to detect low differentiation	High	1.000	**0.248**	0.962
	Low	1.000	**0.262**	0.956
Power to detect high differentiation	High	1.000	1.000	1.000
	Low	1.000	1.000	1.000

(B) Approximate type I error rates (based on 500 replicate simulations) and statistical power based on simulations of differentiation of pollen pools sampled among sites. Numbers in bold denote scenarios with inflated type I error rates.

**Test of Pollen Pool Differentiation Among Sites**	**Genetic Diversity**	**Pollen Pool Differentiation Among Mothers Within Sites**		
		Absence	Low	High
Type I error rate	High	0.066	**0.108**	**0.170**
	Low	0.070	0.076	**0.132**
Power to detect low differentiation	High	1.000	1.000	1.000
	Low	1.000	1.000	1.000
Power to detect high differentiation	High	1.000	1.000	1.000
	Low	1.000	1.000	1.000

(C) Mean simulated values of pollen pool differentiation among site and among mothers within sites, averaged over 500 replicate simulations. Numbers in bold denote the simulated *Φ*-values that most closely match the empirical data.

**Simulated Mean Φ Values (differentiation among sites, differentiation among mothers within site)**		**Genetic Diversity**	**Pollen Pool Differentiation Among Sites**		
			Absence	Low	High
Pollen Pool Differentiation Among Mothers Within Site	Absence	High	0.000, 0.001	0.237, 0.000	0.376, 0.000
		Low	0.000, 0.000	0.117, 0.000	0.278, 0.000
	Low	High	0.000, 0.236	0.227, 0.012	0.342, 0.051
		Low	0.000, 0.116	0.107, 0.012	0.239, 0.052
	High	High	0.000, 0.376	0.167, 0.084	0.199, 0.221
		Low	0.000, 0.278	**0.036, 0.083**	0.074, 0.221

2.7 Supplementary Table 7

Test for presence of null alleles following Brookfield (1996). All loci, corresponding to 71 mothers and 720 seeds, were screened. The 95% confidence interval for each locus is shown.

	Hac_C7	Hac_D1	Hb_B9	Hb_C115	Hac_C114	Hac_B6	Hac_B4	Hac_A103	Hac_A116	Hc_C7	Hc_C126
**Observed frequency**	0.05	0.02	0.1	0.09	0.01	0.21	-0.06	0.1	0.03	0.07	0.02
**Median frequency**	0.05	0.02	0.1	0.09	0.01	0.21	-0.06	0.1	0.03	0.07	0.02
**2.5th percentile**	0.03	-0.01	0.08	0.07	0	0.18	-0.07	0.08	0.01	0.05	0.01
**97.5th percentile**	0.07	0.04	0.13	0.11	0.03	0.24	-0.05	0.12	0.04	0.09	0.04

3 Supplementary Methods

3.1 Simulation of Pollen Pools

We simulated pollen pool differentiation among sites and among mothers within sites to estimate the type I error rates and the statistical power associated with our sampling design and AMOVA models.

We simulated five mother plants with five seeds each across 14 different sites. This resulted in 70 mothers and 350 seeds, similar to the empirical data set. We simulated 10 microsatellite loci for each mother and seed. Microsatellite loci were simulated under two levels of genetic diversity: (1) high, with 10 alleles per locus (100 alleles in total); and (2) low, with 6 alleles per locus (60 alleles in total).

Within each level of genetic diversity, allele frequencies were modified to simulate three levels of pollen pool differentiation among sites: absence of, low, and high levels of differentiation. The order of alleles was randomized for all sites and loci. For the 'absence of pollen pool differentiation among sites' setting, all alleles were sampled with equal probability of 0.1 for the 'high diversity' level and 0.167 for the 'low diversity' level. For the 'low pollen pool differentiation among sites' setting, alleles were sampled with the following probabilities: {0.3, 0.25, 0.2, 0.15, 0.05, 0.05, 0, 0, 0, 0} for the 'high diversity' level; and {0.3, 0.25, 0.2, 0.15, 0.05, 0.05} for the 'low diversity' level. For the 'high pollen pool differentiation among sites' setting, alleles were sampled with the following probabilities: {0.4, 0.3, 0.2, 0.1, 0, 0, 0, 0, 0, 0} for the 'high diversity' level; and {0.4, 0.3, 0.2, 0.1, 0, 0} for the 'low diversity' level.

As we are interested in simulating pollen pool differentiation, all simulations focused on the paternal alleles of the simulated seeds. All simulated mothers had two copies of allele '1' at each locus (homozygotes), while all simulated seeds had one maternal allele '1' at each locus. The second (paternal) allele of each seed was sampled at each locus using the allele frequency distribution described above. We used the same methods described in the main text to subtract the maternal contribution from the genotype of all simulated seeds. Note that we could have simulated only the paternal alleles but decided to include maternal alleles so that we could use the same code as for the empirical data. As maternal alleles are subtracted before the AMOVA, setting them all to allele '1' did not affect the results.

Within each level of genetic diversity, family-specific (all seeds from the same mother) allele frequencies were modified by randomly shuffling the rank order of alleles. Specifically, we randomly shuffled either the first, the first three, or the first five alleles from the site-level allele frequency distribution defined above. With this, we simulated three levels of pollen pool differentiation among mothers within sites: absence of, low, and high levels of differentiation, respectively. Note that shuffling only the first allele is identical to the site-level allele frequencies and thus represents the 'absence of pollen pool differentiation among mothers within sites' setting. For the 'low pollen pool differentiation within sites' setting, the first three alleles were randomly shuffled. For example, if the order of alleles for a site under high genetic diversity was {1, 2, 3, 4, 5, 6, 7, 8, 9, 10}, shuffling the first three alleles might result in the new order {3, 1, 2, 4, 5, 6, 7, 8, 9, 10}, thus changing the allele frequencies of the first three alleles according to the previously defined probability vector (site-level allele frequencies). For the 'high pollen pool differentiation within sites' setting, the first five alleles were randomly shuffled.

In summary, simulations included two levels of genetic diversity, three levels of pollen pool differentiation among sites, and three levels of pollen pool differentiation among mothers within sites. In total we simulated 18 different scenarios, one for each combination of genetic diversity and among- and within-site pollen pool differentiation (Supplementary Table 6A, B, C).

For each scenario, we simulated 500 replicate data sets and tested pollen pool differentiation among sites and among mothers within sites by fitting a hierarchical AMOVA model with 100 permutations. We used simulations under the 'absence of pollen pool differentiation' setting to approximate type I error rates by estimating the proportion of tests with statistically significant results, both among- and within-sites. We used simulations under the 'low' and 'high' pollen pool differentiation settings to approximate statistical power by estimating the proportion of tests with statistically significant results, both among- and within-sites.
